# Osteoclast Immunosuppressive Effects in Multiple Myeloma: Role of Programmed Cell Death Ligand 1

**DOI:** 10.3389/fimmu.2018.01822

**Published:** 2018-08-10

**Authors:** Yu-Tzu Tai, Shih-Feng Cho, Kenneth C. Anderson

**Affiliations:** ^1^LeBow Institute for Myeloma Therapeutics and Jerome Lipper Multiple Myeloma Center, Dana-Farber Cancer Institute, Harvard Medical School, Boston, MA, United States; ^2^Division of Hematology & Oncology, Department of Internal Medicine, Kaohsiung Medical University Hospital, Kaohsiung Medical University, Kaohsiung, Taiwan; ^3^Faculty of Medicine, College of Medicine, Kaohsiung Medical University, Kaohsiung, Taiwan

**Keywords:** multiple myeloma, osteoclast, bone marrow microenvironment, osteoblast, programmed cell death 1, programmed cell death ligand 1, immunotherapy

## Abstract

Immunomodulatory drugs and monoclonal antibody-based immunotherapies have significantly improved the prognosis of the patients with multiple myeloma (MM) in the recent years. These new classes of reagents target malignant plasma cells (PCs) and further modulate the immune microenvironment, which prolongs anti-MM responses and may prevent tumor occurrence. Since MM remains an incurable cancer for most patients, there continues to be a need to identify new tumor target molecules and investigate alternative cellular approaches using gene therapeutic strategies and novel treatment mechanisms. Osteoclasts (OCs), as critical multi-nucleated large cells responsible for bone destruction in >80% MM patients, have become an attractive cellular target for the development of novel MM immunotherapies. In MM, OCs are induced and activated by malignant PCs in a reciprocal manner, leading to osteolytic bone disease commonly associated with this malignancy. Significantly, bidirectional interactions between OCs and MM cells create a positive feedback loop to promote MM cell progression, increase angiogenesis, and inhibit immune surveillance *via* both cell–cell contact and abnormal production of multiple cytokines/chemokines. Most recently, hyper-activated OCs have been associated with activation of programmed cell death protein 1 (PD-1)/programmed cell death ligand 1 (PD-L1) pathway, which impairs T cell proliferation and cytotoxicity against MM cells. Importantly, therapeutic anti-CD38 monoclonal antibodies and checkpoint inhibitors can alleviate OC-induced immune suppression. Furthermore, a proliferation-inducing ligand, abundantly secreted by OCs and OC precursors, significantly upregulates PD-L1 expression on MM cells, in addition to directly promoting MM cell proliferation and survival. Coupled with increased PD-L1 expression in other immune-suppressive cells, i.e., myeloid-derived suppressor cells and tumor-associated macrophages, these results strongly suggest that OCs contribute to the immunosuppressive MM BM microenvironment. Based on these findings and ongoing osteoimmunology studies, therapeutic interventions targeting OC number and function are under development to diminish both MM bone disease and related immune suppression. In this review, we discuss the classical and novel roles of OCs in the patho-immunology of MM. We also describe novel therapeutic strategies simultaneously targeting OCs and MM interactions, including PD-1/PD-L1 axis, to overcome the immune-suppressive microenvironment and improve patient outcome.

## Introduction

Multiple myeloma (MM), a malignancy of plasma cells (PCs), is defined by abnormal growth of malignant PCs within the bone marrow (BM), resulting in excessive monoclonal immunoglobulin in the blood and urine, impaired renal function, and repeated infections in patients (1). Moreover, osteolytic bone disease is a central hallmark of MM, which severely impacts quality of life in >80% of patients (2, 3). Specifically, osteoclast (OC)-mediated lytic bone destruction remains a cause of major morbidity in MM. In the past two decades, the introduction of autologous stem-cell transplantation and the availability of novel agents with different mechanisms of action including proteasome inhibitors (e.g., bortezomib, carfilzomib, ixazomib) and immunomodulatory drugs (IMiDs) (e.g., thalidomide, lenalidomide, pomalidomide) have revolutionized the therapeutic strategies for MM and significantly prolonged overall survival of patients (4–7). However, cure is rarely achieved due to the development of drug resistance and persistence of minimal residual disease. Thus, there is unmet need for innovative treatment modalities to eradicate residual tumor clones and effectively prevent disease relapses, as well as enhance overall anti-MM immunity.

Recently, immunotherapies have showed significant clinical activities not only against malignant, PCs but also potentially relieving the immunocompromised status in MM. Currently, a variety of immunotherapeutic strategies are under intensive preclinical and clinical development, including monoclonal antibodies (mAbs), chimeric antigen receptor T (CAR T) cells, immune checkpoint inhibitors, and as well as cancer vaccines (8). Following the approval of the first two mAbs daratumumab targeting CD38 and elotuzumab targeting SLAMF7 by FDA in late 2015 for the treatment in relapse and refractory MM (RRMM), multiple combination trials of these two mAbs are ongoing (8, 9). Excitingly, daratumumab has also shown clinical responses in newly diagnosed MM patients (9). Another therapeutic anti-CD38 mAb isatuximab, unlike daratumumab, can directly kill MM cells with p53 mutations and in the absence of effector natural killer (NK) cells *in vitro* (10). Indeed, isatuximab, when combined with lenalidomide or pomalidomide plus dexamethasone, also demonstrated significant activity in heavily treated RRMM (11, 12). Isatuximab is currently undergoing studies for the treatment of relapsed and previously untreated MM patients, pursuing FDA approval. Most importantly, more than a dozen targeted immunotherapies besides CD38 and SLAMF7 mAbs, alone or in combinations with current or emerging anti-MM therapies with different mechanisms of actions, have already entered clinical investigations.

Accumulating data for the past two decades has confirmed that the BM microenvironment plays a crucial role in the pathogenesis and recurrence of MM (13, 14). Malignant PCs in the MM BM are in close contact with non-myeloma cells, including bone marrow stromal cells (BMSCs) (13, 15), osteoclasts (OCs) (16–20), myeloid-derived suppressor cells (MDSCs) (21, 22), tumor-associated macrophages (TAMs) (23), regulatory T-cells (Treg) (21, 24, 25), plasmacytoid dendritic cells (pDC) (26), and regulatory B-cells (Breg) (27). These BM accessory cells, alone or in collaboration with others, support the initiation, progression, and re-occurrence of MM. They further influence treatment responses and may promote clonal evolution of malignant PC clones to adapt to the immune microenvironment and escape immune surveillance. For example, MM cells increase their proliferation upon adherence to BMSCs and become resistant to dexamethasone treatment (13, 28). Cytotoxic effects of some conventional drugs, i.e., dexamethasone, melphalan, as well as antibody-mediated cellular cytotoxicity against MM cells are reduced in the presence of BMSCs (13, 29).

**Figure 1:**
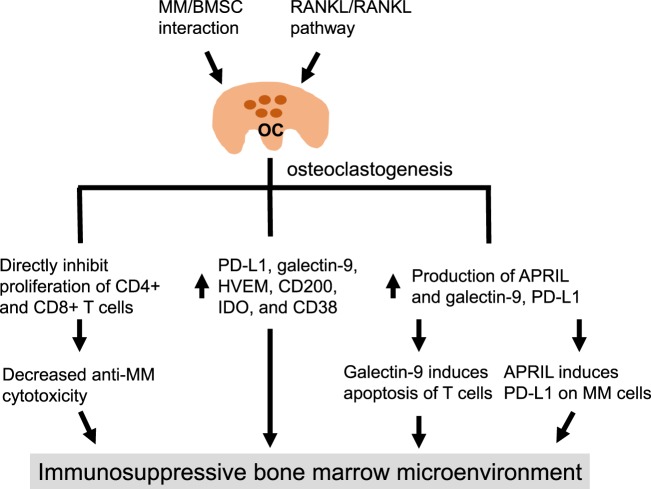
Osteoclasts create an immunosuppressive microenvironment in multiple myeloma (MM). In MM, the interaction of MM cells and bone marrow stromal cells induces production of various cytokines and growth factors, as well as activates RANK/receptor activator of nuclear factor-κB (NF-κB) ligand pathway, to promote the differentiation and expansion of OCs from CD14+ OC precursors. OCs can directly inhibit proliferation of activated CD4+ and CD8+ effector T cells, thereby reducing their numbers and leading to decreased MM cell lysis. The expression of multiple immune checkpoint molecules on OCs is increased during osteoclastogenesis. Furthermore, the secretion of galectin-9 and APRIL is significantly augmented during OC formation, resulting in apoptosis of T cells, i.e., mediated by galectin-9, and enhanced programmed cell death ligand 1 expression on MM cells, i.e., mediated by APRIL, IL-6. APRIL, a proliferation-inducing ligand; BMSC, bone marrow stromal cell; HVEM, herpesvirus entry mediator; IDO, indoleamine 2, 3-dioxygenase.

Among other abovementioned cells, hyperactive OCs cause osteolytic bone diseases affecting almost every MM patient, thereby making them a potential novel cellular target for novel therapeutics. OCs, critical mediators of bone absorption, are large cells with multiple nuclei derived from CD14+ lineage myeloid cells (i.e., monocyte, macrophage) under the influence of several OC-activating cytokines produced by multiple BM accessory cells. Among many OC-stimulating cytokines, macrophage-colony-stimulating factor (M-CSF) and receptor activator of nuclear factor-κB (NF-κB) ligand (RANKL) are two essential OC-differentiation factors during osteoclastogenesis. Traditionally, OCs are known to play a vital role in maintenance of bone metabolism by counteracting osteoblasts (OBs). In contrast to OBs, which produce and secrete matrix proteins and transport mineral into the matrix for bone formation, OCs are responsible for bone degradation by breaking down tissues. In addition to inducing growth and survival of MM cells, OCs are capable of regulating growth of other BM cells, such as hematopoietic stem cells and B cell progenitors (30–32). Moreover, a close crosstalk exists between skeletal and immune systems, termed osteoimmunology, since several regulatory molecules are shared by these two systems (33–35). Most recently, OCs have been further associated with maintenance of immunosuppressive MM BM microenvironment *via* induction and secretion of several immune checkpoint proteins from OCs in close contact with MM cells (20) (Figure 1).

Programmed cell death ligand 1, also known as cluster of differentiation 274 (CD274) or B7 homolog 1 (B7-H1), is a 40 kDa type 1 transmembrane protein encoded by the CD274 gene located in the 9p24.1 region with the full length of cDNA 870 bp in man (36, 37). Following binding to its cognate receptor programmed cell death protein 1 (PD-1) (CD279) expressed on activated T cells, B cells, NK cells, and monocytes, the PD-1/PD-L1 pathway inhibits immune activation by triggering the phosphatases that deactivate signals emanating from the T cell receptor (38–40). Specifically, the engagement of PD-L1 with PD-1 on activated T cell leads to T cell dysfunction, exhaustion, neutralization, and production of interleukin-10 (IL-10) (41, 42). PD-L1 also interacts with B7-1 (CD80) on activated T cells, which in turn downregulates T cell activity (43). This important checkpoint pathway has been associated with autoimmune disease, infection, and cancer (37, 44–46).

In the tumor microenvironment, PD-1/PD-L1 pathway performs a vital role in tumor progression and survival by escaping tumor neutralizing immune surveillance. PD-L1 is expressed on various tumor cells and antigen-presenting cells (APCs) (41). PD-L1 overexpression on tumor cells is further associated with higher risk of cancer progression and poor clinical outcome (47–49). Importantly, immune checkpoint inhibitors targeting PD-1/PD-L1 have generated groundbreaking and durable responses in a broad spectrum of advanced solid tumors (50) and blood cancers including B-cell lymphomas (51, 52). In MM, PD-1/PD-L1 is also activated and associated with immunocompromised status and drug resistance (53, 54), supporting the development of new treatments targeting this pathway in MM (55). Despite inconclusive early clinical reports (51, 55), this important immune checkpoint pathway may still represent one of the novel strategies with potential anti-MM activities targeting defective immune effector cells, when combined with current and emerging therapies for MM.

We here summarized mechanisms of myeloma bone diseases and the novel functional characterization of OCs in the immunosuppressive BM microenvironment in MM *via* PD-1/PD-L1 pathway. Also included are effects of various current and emerging anti-MM treatments on OCs, other cellular subtypes associated the MM bone disease, and immune cells in the BM. Finally, we discuss the novel strategies for immune-therapies targeting OC function and PD-1/PD-L1 pathway in combination with other MM treatments to further overcome OC-induced immune suppression and prolong overall treatment responses.

## Myeloma Bone Disease: Clinical Manifestation

The cells in skeletal system including OBs, OCs, and osteocytes closely communicate with each other to maintain the balance of bone metabolism. OBs provide essential signals, M-CSF, RANKL, and other co-stimulatory factors, to promote the differentiation of myeloid lineage precursors of OCs (56). However, this balance is significantly disturbed in the majority of MM patients, in whom OCs are highly activated accompanied with little or no OB activity (2). Eventually, increased bone-degrading effects accelerate osteoporosis and the development of lytic bone lesions, shown as characteristic “punched-out” lesions on skeletal X-ray (57, 58).

Clinically, approximately 80% of MM patients have radiologic evidence of bone involvement, and 90% have osteolytic manifestations including generalized osteopenia or discrete lytic lesions over the course of disease (16, 59). The most commonly involved sites include vertebral bodies (49%), skull (35%), pelvis (34%), and ribs (33% of patients) (2, 3, 60). Patients with MM bone disease may suffer from skeletal-related events (SREs) including pain, pathological fractures, spinal cord compression, and hypercalcemia. Furthermore, these SREs may increase mortality, decrease quality of life, and result in an adverse outcome (58, 61).

## Myeloma Bone Disease: Major Cellular and Molecular Mechanisms

The mechanisms of MM-related bone disease involve overactivation of OCs and inhibition of OBs *via* complicated interactions between various BM cells and cytokines secreted by them (2). The contact between MM cells and BMSCs significantly increases activity and accelerates differentiation of OCs, while inhibiting the growth of OBs (15, 62). For example, the binding of surface VLA-4 (α_4_β_1_ integrin) on MM cells to VCAM-1 on BMSCs induces production of cytokines, which favor bone absorption including: RANKL, M-CSF, IL-1, and IL-6 by BMSCs; and OC-activating factors including macrophage inflammatory protein-1α/β (MIP-1α/β), IL-3, stromal-derived factor-1α, and tumor necrosis factor α (TNF-α) by MM cells(63–70). In addition, adhesion between MM cells and BMSCs promotes secretion of B-cell activation factor (BAFF), which also promote growth of MM cells (71, 72) and RANKL-independent proliferation of OCs (72). In parallel, p38 mitogen-activated protein kinase signaling pathway is activated upon MM cell adherence to BMSCs, leading to more secretion of MM cell-supportive factors IL-6 and vascular endothelial growth factor (VEGF), in addition to induction of OC-activating factors (i.e., IL-11, RANKL, MIP-1α) (73). Moreover, IL-6 secretion by BMSC enhances expression and secretion of matrix metalloproteinase-13 (MMP-13) in MM cells (74). MMP-13, in turn, promotes fusion of OCs and bone absorption. Simultaneously, activated OCs support proliferation of MM cells by secreting more factors including annexin-II, osteopontin (OPN), IL-6, IL-10, insulin growth factor-1, BAFF, and a proliferation-inducing ligand (APRIL) (13, 20, 75–78).

In contrast, the expansion and activation of OBs is significantly blocked in MM bone disease due to increased secretion of OB inhibitory factors including: dickkopf-1 (Dkk-1), soluble frizzled-related protein 2 (sFRP2), sFRP3, IL-3, IL-7, growth factor independence-1 (gfi1), hepatocyte growth factor, activin A, sclerostin, and TNF-α (2, 62, 79–84). These factors directly and indirectly block proliferation and differentiation of OBs, impairing mineral deposition and bone regeneration. In addition, osteoprotegerin (OPG), a soluble decoy receptor of RANKL, is produced by OBs and inhibits OC activation under normal physiological conditions. OPG levels are significantly decreased in MM bone disease (85), associated with reduced OB number. Defective bone formation due to decreased proliferation and differentiation of OBs induced by MM cells, along with reduced levels of OC inhibitory cytokines produced by OBs, further augments OC formation and induction of osteolytic bone destruction.

In terms of signaling transduction cascades, the RANK/RANKL pathway critically regulates MM-induced bone lesions since several of the abovementioned OC-activating factors are induced *via* this pathway. RANKL is detected on the surface of MM cells and elevated in MM patients compared with health individuals and patients with monoclonal gammopathy of undetermined significance (MGUS) (86, 87). Concurrently, increased OCs induced by RANKL activate dormant MM cells (32). In fact, higher RANKL expression is associated with more severe bone disease and poorer clinical outcome (86, 88). In addition, MM cells express mRNA encoding the isoform of soluble RANKL (sRANKL), which directly promotes activation of OCs (89). Significantly, sRANKL is elevated in MM patients and closely related to generalized bone loss (90, 91).

Further studies on OC-gene expression profiling identify genes coding for 4 CCR2-targeting chemokines and genes coding for MM growth factors to be highly expressed by MM OCs (92). Specifically, higher CCR2 expression in MM cells is correlated with increased bone lesions, and CCR2 chemokines activate mitogen-activated protein kinase (MEK) pathway to support growth of MM cells (92). These results implicate the MEK1/2 signaling cascade (93), which is significantly induced by M-CSF and RANKL, in the pathogenesis of MM bone disease(17, 18, 94).

## OCs in the MM BM Microenvironment

The suppression of the host immune system is a critical step in the progression of many cancers, including MM. The interaction of MM cells and surrounding cells promotes production of immunosuppressive cytokines, growth of immune-suppressive cell populations, and suppression of the anti-MM ability of normal immune cells. For example, IL-6 and IL-10 levels are increased in the serum samples of MM patients, and both cytokines promote MM cell growth and survival in an autocrine and paracrine fashion. These two cytokines are also critical in MM-related immunosuppression, since IL-10 has potent immunosuppressive ability by inhibiting production of pro-inflammatory interferon-γ (IFN-γ) and TNF-α in immune effector cells (95), and IL-6 has been linked to impaired NK cell activity (96). Furthermore, the pro-osteoclastogenic LIGHT/TNFSF14 was recently linked to MM-bone disease (97). At the cellular level, inhibitory immune T regulatory cells (Tregs), B regulatory cells, and pDCs are significantly increased in the BM of the patients with active MM (24, 26, 27). In parallel, MM cells induce the development of myeloid-derived suppressor cells (MDSCs), which in turn support proliferation of MM cells by promoting proliferation of Tregs and suppressing T-cell-mediated immune responses (22, 98). Importantly, MDSCs induced by MM cells can further differentiate into mature OCs capable of inducing bone lysis, which further links immune suppression and hyper-active bone lysis activity of MDSCs in MM progression (99). Furthermore, the increased percentage of circulating pre-OCs have been described in MM (100, 101).

The MM BM microenvironment is also characterized by increased angiogenesis, which further suppresses anti-MM immunity. Specifically, contact of MM cells and OCs enhances angiogenesis and production of angiogenic factors (VEGF and OPN), which in turn promote the expansion of OCs by vascular endothelial cells (102). Both VEGF and OPN have been shown to directly induce proliferation of MM cells. In addition, increased OC formation by stimulation of RANKL or parathyroid hormone-related protein promotes angiogenesis *via* induction of MMP-9, a potent angiogenic factor secreted by OC mediating RANKL-induced angiogenesis. In contrast, OPG inhibits formation of OCs and decreases formation of new vessels (103).

**Figure 2:**
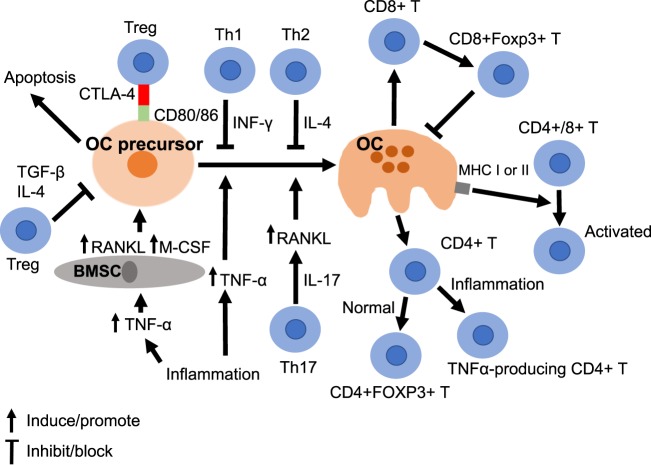
Osteoclasts crosstalk with immune cells. The differentiation of OCs from its precursor (OC precursor) is mediated by multiple cytokines. For example, inflammation induces production of tumor necrosis factor α (TNFα), which activates OC formation directly or indirectly *via* BMSC. Another immune cell, the Th17 cell, which produces IL-17, also stimulate OCs *via* upregulation of receptor activator of nuclear factor-κB (NF-κB) ligand. The process of OC differentiation can be inhibited by INF-γ and IL4 produced by Th1 and Th2 cells, respectively. In parallel, T regulatory cells (Tregs) can inhibit OC precursors by secretion of TGF-β and IL-4. CTLA 4 expressed on Tregs can bind to CD80/86 on OC precursors and further influence the fate of OC precursors. OCs can activate several immune cells. First, OCs induce formation of CD8+FOXP3+ T cells, which in turn inhibit OCs. Second, OCs can act as antigen-presenting cells to promote immune response of CD4 or CD8 T cells. Third, OCs can induce differentiation of CD4+ T cell to TNFα-producing cells or CD4+FOXP3+ T cells, dependent on the surrounding microenvironment. BMSC, bone marrow stromal cell; IL, interleukin; INF, interferon; M-CSF, macrophage-colony-stimulating-factor; TGF, tumor growth factor; TNF, tumor necrosis factor; Treg, regulatory T cell.

Most recently, OCs have been shown to significantly block T cell proliferation and cytotoxicity in MM cells (Figure 2). The expression of several immune checkpoint molecules on OCs, including PD-L1, galectin-9, herpesvirus entry mediator, CD200, T-cell metabolism regulators indoleamine 2, 3-dioxygenase (IDO), and CD38, is significantly enhanced during OC formation *in vitro* (20) (Figure 1). Meanwhile, the secretion of galectin-9 and APRIL by OCs is significantly increased. Galectin-9 significantly induces apoptosis of T cells, and APRIL further induces expression of PD-L1 on MM cells mainly *via* MEK/ERK pathway. Significantly higher expression of PD-L1 was observed on OCs than MM cells, which was linked to profound inhibition of T cell activation to lyse MM cells. Importantly, the inhibition of T cell activation can be repaired using blocking PD-L1 or anti-CD38 monoclonal antibody (20), suggesting potential clinical development of these mAbs, alone and in combination, to overcome the immunosuppressive MM BM milieu.

## OCs in Osteoimmunology

The skeletal and immune systems closely interact, since cytokines produced by lymphocytes significantly affect bone homeostasis. Among cells in these two systems, OCs significantly regulate intricate cytokine and cellular networks, as described above and in Figure 2. OCs interact not only with various BM cytokines but also control differentiation and expansion of multiple immune subsets. For example, inflammation or immune-related cytokines like TNF-α, IL-1, and IL-6, are associated with bone absorption (104–107). In autoimmune diseases like rheumatoid arthritis, production of cytokines (TNF-α, IL-1, IL-6, IL-17) is significantly increased in synovium and pannus, which may directly affect bone by upregulating OC activities at sites of articular erosion (108). In fact, TNF-α induces activation of OCs indirectly by enhancing the expression of RANKL and M-CSF in BMSCs or directly by interacting with OC precursors (109).

As for the interaction between OCs and immune cells, activated CD3+ or CD4+ T cells with RANKL expression support differentiation of OC *in vitro* (110, 111). A subset of CD4+ T cells (Th17), which produces IL-17 could upregulate RANKL and promote differentiation of OCs by the effect of IL-17 on BMSCs and OCs (112). T cells also produce IL-7, which can promote formation of OCs by upregulating RANKL (113). In addition, activated T cells secrete soluble RANKL (sRANKL), which is correlated with the formation of OCs and bone loss (114, 115). On the other hand, the activation of OCs can be downregulated by IFNγ and IL-4 secreted by Th1 and Th2 cells, respectively. IFN-γ produced by T cells significantly suppresses differentiation of OCs by interfering with the RANKL–RANK pathway, including degradation of downstream molecules such as tumor necrosis factor receptor-associated factor 6 (TRAF6) (116).

On the other hand, human OCs can function as APCs by expressing class I and II MHC molecules and co-stimulatory molecules to in turn activate both CD4+ and CD8+ T cells (117). In a mouse model study, expression of RANKL was detected on the surface of activated CD4+ and CD8+ T cells (118). Conversely, inhibition by a RANKL inhibitor suppresses activation of T cells, suggesting the role of RANK/RANKL pathway in T cell activation. Meanwhile, OCs are capable of inducing differentiation of CD8+ T cells into FoxP3+ CD8+ Tregs, which not only decrease antigen-specific T cell proliferation but also suppress bone resorption by forming a negative feedback loop (119–123). In a similar fashion, adoptive transfer of CD4+CD25+ Tregs into T-cell deficient mice enhances bone mass formation accompanied by decreased OC numbers, partially mediated by IL-4 and IL-10 (124). In addition, isolated human Tregs suppress OC differentiation *via* the secretion of TGF-β and IL-4 (125). CD4+CD25+Foxp3+ Treg can also inhibit differentiation of OCs by cytotoxic T lymphocyte antigen 4 (CTLA4) in a cell-to-cell contact-dependent manner (126, 127). Specifically, CTLA4 on Tregs downregulates proliferation of OCs by binding to CD80/86 on OC precursors (128). The engagement of CD80/86 by CTLA-4 in OC precursors activates IDO, which in turn further degrades tryptophan and induces apoptosis of OC precursors.

A recent study in a mouse model showed that OCs derived from normal BM can induce CD4+FoxP3+ regulatory T cells (129). On the contrary, OCs can induce TNFα-producing CD4+ T cells in an inflammatory bowel disease mouse model (129). All these findings suggest that OCs not only play a role in immune suppression, but also serve as true APCs depending on the origin and environment.

## PD-L1 Expression on MM Cells and OCs

Programmed cell death protein 1/PD-L1 pathway contributes to tumor progression and survival by escaping tumor neutralizing immune surveillance in the tumor microenvironment (130). PD-L1 has been linked to the maintenance of Tregs, which are associated with suppression of antitumor immune response (131). The expression of PD-L1 on tumor cells can be enhanced by IFNγ secreted by activated cytotoxic T cells in the tumor microenvironment, thereby downregulating antitumor immunity (132). In addition, PD-L1 expression can be altered by extrinsic factors like inflammatory cytokines, which induce signaling cascades including MEK/ERK, PTEN, mTOR, or PI3K pathways (133–135).

In MM, PD-L1 is expressed on PCs isolated from patients with MM and MM cell lines, but not on normal PCs (20, 133, 136–138). The percentages of PD-L1 + PCs are higher on MM and smoldering MM than MGUS (133). Increased PD-L1 levels in MGUS patients is further linked to a higher risk of progression to clinical MM (139). PD-L1 expression on MM cells is enhanced following stimulation of IFNγ *via* activation of MYD88, TRAF6, and MEK/ERK signaling pathways; conversely, MEK1/2 inhibitors partially block IFNγ-induced PD-L1 upregulation (20, 133). BMSCs also induce expression of PD-L1 on MM cells by production of IL-6 *via* signal transducer and activator of transcription 3, MEK1/2, or Janus kinase 2 (140, 141). In addition, MM cells with PD-L1 expression are correlated with higher proliferation rate and higher expression of BCL-2 and FasL than MM cells without PD-L1 expression. Moreover, the interaction between PD-L1 on MM cells and PD-1 not only inhibited tumor-specific cytotoxic T cells but also promoted drug resistance in myeloma cells through the PI3K/AKT signaling cascade (53). Importantly, higher serum level of soluble PD-L1 in MM patients is associated with shorter progression-free survival (142).

Programmed cell death ligand 1 is expressed on multiple immune cell subsets in the MM BM microenvironment, including pDCs (137, 143), MDSCs (141), and OCs (20). Specifically, PD-L1 on pDCs is overexpressed in 81% of cases (143). Expression of PD-L1 is significantly higher on the CD141+ subset, which regulates immune response of CD8+ T cells, than on the CD141-negative CD4+ T cells. PD-L1 on immunosuppressive MDSCs is increased in patients with RRMM compared with newly diagnosed MM (141). Significantly, blockade of PD-1/PD-L1 pathway inhibits MDSC-mediated growth of MM cells. Furthermore, BM mesenchymal stem cells promote proliferation and reduce apoptosis of MM cells by suppressing T-cell immune responses *via* PD-1/PD-L1 pathway (144).

**Figure 3:**
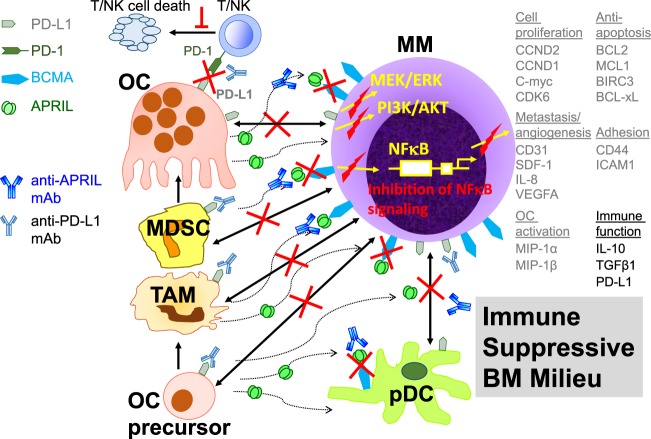
Targeting a proliferation-inducing ligand (APRIL) and programmed cell death ligand 1 (PD-L1) to overcome OC-mediated immune suppression in the multiple myeloma (MM) BM milieu. APRIL is secreted by myeloid lineage cells including OCs, OC precursors, tumor-associated macrophages, and MDSCs, in the BM. MDSCs induced by MM cells further differentiate into functional OCs. Besides the induction of critical downstream targets (listed in gray on the right), APRIL induces PD-L1 on MM cells *via* BCMA, a specific MM antigen. This positive feedback loop between MM cells and MM-supporting cells, coupled with increased PD-L1 expression, further inhibits effector-mediated MM cell lysis *via* binding to programmed cell death protein 1 (PD-1) on activated T and natural killer (NK) effector cells. Blocking PD-1/PD-L1 and APRIL monoclonal antibodies prevent these effects and may mitigate immune suppression in MM. Adapted from Ref. (20, 145). TAM, tumor-associated antigen; MDSC, myeloid-derived suppressor cells; BCMA, B cell maturation antigen; T/NK, immune effector cells; pDC, plasmacytoid dendritic cell. Red lines indicate inhibition in the presence of these blocking monoclonal antibodies; double arrow lines depict interactions.

Furthermore, OCs induce expression of PD-L1 on MM cells in an APRIL-dependent manner *via* binding of two APRIL receptors (BCMA and TACI), which are highly expressed on MM cells (20, 145) (Figure 3). Since OCs are the key physiological source of APRIL production in the BM microenvironment, these results further provide evidence of a positive feedback loop between OCs and MM cells in promoting PD-L1-mediated immunosuppression in MM. Meanwhile, increased PD-L1 expression on OCs further enhances immunosuppression by promoting the binding of PD-1 on T cells and inducing dysfunction and apoptosis of effector T cells (20).

Some current and emerging anti-MM agents can affect the expression of PD-L1 on MM cells (Table 1). For example, proteasome inhibitors, oncolytic reovirus, and a histone deacetylase inhibitor 6 (HDAC) inhibitor have been shown to enhance PD-L1 expression on MM cells (146–148). On the other hand, lenalidomide and MEK1/2 inhibitors, as well as APRIL blocking reagents, reduce PD-L1 induction on MM cells (20, 133, 141, 145, 149). These findings support further investigations targeting PD-L1 in MM.

**Table 1:** Summary of trials of programmed cell death ligand 1 (PD-L1) inhibitors and treatment, which modulates PD-L1 expression.

Agents	Effect on PD-L1 in multiple myeloma (MM) cells	Clinical trials in MM
Atezolizumab	Direct inhibition	Alone or in combination with an IMiD and/or daratumumab in relapse and refractory MM (RRMM) patients, phase 1 (NCT02431208). Status: recruiting
Durvalumab	Direct inhibition	Monotherapy or in combination with pomalidomide with or without dexamethasone in RRMM patients, phase 1 (NCT02616640). Status: active, not recruiting Combination of durvalumab with lenalidomide with or without dexamethasone in newly diagnosed MM patients, phase 1 (NCT02685826). Status: active, not recruiting Combination of durvalumab with daratumumab with or without pomalidomide and dexamethasone in RRMM patients, phase 2 (NCT02807454). Status: active, not recruiting
Proteasome inhibitor	Upregulation	Bortezomib with oncolytic reovirus and dexamethasone in RRMM patients (NCT02514382). Status: recruiting
Oncolytic reovirus	Upregulation	Bortezomib with oncolytic reovirus and dexamethasone in RRMM patients (NCT02514382). Status: recruiting Combined with lenalidomide or pomalidomide in RRMM patients, phase 1 (NCT03015922). Status: recruiting
HDAC6 inhibitors	Upregulation in MM cells	Ricolinostat (ACY-241): Combination with pomalidomide and dexamethasone in RRMM patients, phase 1b/2 (NCT01997840). Status: active, not recruiting
MEK1/2 inhibitor	Downregulation	Binimetinib with encorafenib in RRMM patients with BRAFV600 E or BRAFV600K mutation, phase 2 (NCT02834364). Status: recruiting
BTK inhibitor	Downregulation	Ibrutinib with carfilzomib and dexamethasone in RRMM patients, phase 1/2 (NCT01962792). Status: active, not recruiting Ibrutinib with pomalidomide, and dexamethasone in RRMM patients: phase 1/2 (NCT02548962). Status: active, not recruiting Ibrutinib, bortezomib, and dexamethasone in RRMM patients, phase 2 (NCT02902965). Status: active, not recruiting
A proliferation-inducing ligand (APRIL) inhibitor	Downregulation	BION-1301 in RRMM patients, phase 1/2 (NCT03340883). Status: recruiting
APRIL CAR T cells	Downregulation	RRMM patients, phase 1/2 (NCT03287804). Status: recruiting
BCMA CAR T cells	Downregulation	bb2121 in RRMM patients, phase 1 (NCT02658929). Status: active, not recruiting. bb2121 in RRMM patients, phase 2 (NCT03361748). Status: recruiting. Anti-BCMA CAR-T for heavily pretreated MM patients, phase 1 (NCT02215967). Status: active, not recruiting. Combination of anti-BCMA CAR-T with lenalidomide in RRMM patients, phase 1 (NCT03070327). Status: recruiting.

## Targeting PD-1/PD-L1 Pathway with Various Current and Novel MM Treatments

### Preclinical Studies

The combination of hematopoietic stem cell transplantation with whole-cell vaccination and PD-L1 blockade significantly improves the survival of MM-bearing mice (136). In another study where anti-MM activity is mainly mediated by pre-activated T cells, the combination of anti-PD-L1 inhibitor plus lymphodepletion by sublethal dose of radiation augments T-cell-mediated anti-MM effect and significantly improves survival of mice (54).

A combination of PD-1/PD-L1 blockade with IMiDs was also investigated in a study where NK cells or T cells were cocultured with CD138+ tumor cells isolated from MM patients and treated with PD-1or PD-L1 inhibitor, alone or together, and with lenalidomide (141). The immune checkpoint blockade by PD-1 or PD-L1, or PD-1/PD-L1 inhibitor combination, induced effector cell-mediated anti-MM cytotoxicity (137, 141). The expression of PD-1 and PD-L1 on effector cells and MM cells was downregulated by lenalidomide. Lenalidomide further augments anti-MM cytotoxicity mediated by checkpoint blockade dependent on NK and T effector cells. In addition, PD-1 inhibitor enhances production of INF-γ and granzyme B from NK cells against MM cells. Treatment with lenalidomide further upregulates PD-1 inhibitor’s enhancement of NK-cell lysis of MM cells (150).

When combined with HDAC6 inhibitor, anti-PD-L1 antibody can trigger even higher MM cell killing mediated by NK and cytotoxic T cells, compared with killing in the presence of either agent alone (148). In addition, oncolytic reovirus enhances expression of PD-L1 on MM cells and augments the anti-MM effect of anti-PD-L1 inhibitor (147). Furthermore, since T-cell dependent bispecific antibody (TDB) induces the expression of PD-1 on CD8+ T cells following the engagement of T cells and target MM cells, treatment with PD-L1 inhibitor could enhance anti-MM activity of MM targeted TDB, as recently shown using an anti-FcRH5/CD3 TDB (151).

### Clinical Studies

In a phase 1b study, monotherapy with PD-1 inhibitor nivolumab was administered in RRMM patients (152); however, no obvious disease regression was observed. The preliminary data from a phase 1 study, which investigated anti-PD1 antibody pembrolizumab in combination with lenalidomide and low-dose dexamethasone in patients with RRMM showed high response rate (76%) (153). Another phase 2 trial combining pembrolizumab, pomalidomide, and dexamethasone in RRMM patient also showed high response rate (60%) (154). This study further showed that higher PD-L1 expression on MM is linked to better progression-free survival. Importantly, however, there were more deaths in phase III trials in the cohorts comparing lenalidomide or pomalidomide with dexamethasone together with pembrolizumab than in patients treated with lenalidomide or pomalidomide with dexamethasone, which has curtailed the development of IMiD pembrolizumab combinations.

Regarding clinical trials of PD-L1 antibodies, single agent durvalumab or the combination of durvalumab with lenalidomide (NCT02685826) is being evaluated in patients with newly diagnosed MM. Durvalumab, alone or combined with pomalidomide (NCT02616640); as well as durvalumab in combination with daratumumab or in combination with pomalidomide, dexamethasone, and daratumumab (NCT02807454) are also clinical trials in RRMM patients. However, these trials are currently not actively recruiting patients for the time being due to the abovementioned safety concern. Nonetheless clinical trials of another anti-PD-L1 antibody, atezolizumab are ongoing in patients with RRMM (NCT02431208).

In addition to direct blockade of PD-1/PD-L1 by PD-1 or PD-L1 inhibitor, novel therapeutic interventions, which modulate the expression of PD-L1 on MM cells are under clinical evaluation in RRMM patients, including the combination of oncolytic reovirus with lenalidomide or pomalidomide (NCT03015922), or oncolytic reovirus with bortezomib and dexamethasone (NCT02514382). Moreover, HDAC6 or MEK inhibitors are also under clinical investigation to potentially modulate expression pattern of PD-1 and PD-L1. The studies of PD-L1 inhibitors or PD-L1 modulators are listed in Table 1.

## Therapeutic Interventions Targeting OCs in MM Therapies

Many novel agents have been under evaluation not only for their direct anti-MM activity but also their abilities to abrogate MM-supporting activities in the BM microenvironment, including targeting of OCs. They include bortezomib and IMiDs, which are already standard anti-MM therapies, as well as several novel agents showing promising results in preclinical studies (Table 2).

**Table 2:** Summary of therapeutic agents targeting osteoclasts (OCs) and other cells associated with multiple myeloma bone disease.

Agents	Mechanisms	Reference
Proteasome inhibitor	Induce apoptosis and block differentiation of OCs Increase differentiation of OB Reduced level of DKK-1 and receptor activator of nuclear factor-κB (NF-κB) ligand (RANKL)	(94, 155, 157, 159, 160)
Immunomodulatory drugs	Targeting PU.1 and pERK to inhibit formation of OC Normalize RANKL/osteoprotegerin ratio	(162, 163)
Bisphosphonate	Induce OC apoptosis but protect OB from apoptosis Block differentiation and maturation of OC	(166, 167)
RANKL inhibitor	Decrease OC formation and activity. Minimal or stimulatory effects on OB.	(18, 168, 169)
BTK inhibitor	Suppress bone resorption and differentiation of OCs Inhibit secretion of multiple cytokines and chemokines from OCs and bone marrow stromal cells	(93, 94)
Anti-CD38 antibody	Inhibition of OC formation and bone resorption Overcome the inhibition of T-cell proliferation blocked by OCs Inhibit immune checkpoint molecules on OCs	(20, 173)
Programmed cell death protein 1/programmed cell death ligand 1 antibody	Block OC-mediated inhibition in T-cell activation and proliferation	(20)

Bortezomib, as a proteasome inhibitor, not only has direct anti-MM activity, but also targets cells associated with MM bone disease. Bortezomib induces dose-dependent growth inhibition and apoptosis, as well as blocks differentiation, of OCs. It further decreases the resorption capacity of mature OCs, reduces the total number of functional OCs, and increases differentiation of OBs (155–157). In addition to the induction of differentiation and growth of OBs, therapeutic proteasome inhibitors bortezomib and carfilzomib promote bone nodule formation, associated with reduced levels of DKK-1 and RANKL (158–160). Bortezomib preferentially induces differentiation of mesenchymal stem/progenitor cells to OBs by regulating expression of the bone-specifying transcription factor runt-related transcription factor 2 in a mouse model (161).

Immunomodulatory drugs inhibit formation of OCs by inhibiting PU.1 and pERK (2, 162). Cathepsin K, an important molecule in bone collagen matrix resorption, and the serum level of RANKL and RANKL/OPG ratio are significantly reduced in MM patients receiving lenalidomide treatment. Furthermore, lenalidomide and pomalidomide normalize RANKL/OPG ratio and inhibit upregulation of RANKL by downregulating adhesion molecules on MM cells (163).

Bisphosphonate is routinely used in MM bone disease treatment to reduce risk of skeletal events (164, 165). Bisphosphonate has high affinity for bone mineral surfaces at sites of active bone remodeling by OCs. It induces apoptosis of OCs while protecting OBs from apoptosis, in addition to blocking differentiation and maturation of OCs (2, 166, 167).

Denosumab (AMG165), a fully human monoclonal antibody (IgG2), blocks the binding of RANKL to its receptor expressed on OCs and their precursors, leading to decreased OC activity and inhibition of bone resorption, followed by increased bone mass and strength (168, 169). Denosumab reduces bone resorption, increases mass of cortical and cancellous bone, and improves the microstructure of trabecular bone (170). A phase 3 clinical trial in MM has shown that denosumab is not inferior to zoledronic acid, the bisphosphonate most commonly used to reduce skeletal-related event in newly diagnosed MM patients (3, 171).

The development and integration of anti-CD38 monoclonal antibody is an important milestone in MM immunotherapy. In addition to MM cells, CD 38 is also expressed on normal PCs, NK cells, monocytes, early OC progenitors, and OCs, but not on the surface of stromal and osteoblastic cells (172, 173). Daratumumab inhibits OC formation and bone resorption (173). The inhibition of T-cell proliferation caused by OCs is partially overcome by anti-CD38 monoclonal antibody isatuximab (20) *via* inhibition of multiple immune checkpoint molecules expressed on OC. Since anti-PD-L1 partially overcomes inhibitory effects of OCs on T-cell activation and proliferation, these results suggest potential therapeutic benefit of combining CD38 and PD-1/PD-L1 mAbs to block OC-induced immunosuppression in MM.

## Perspectives and Conclusion

Programmed cell death protein 1/PD-L1 pathway plays a critical role in the immunosuppressive tumor microenvironment in MM. As PD-L1 is overexpressed in MM patient cells and other cells associated with immunosuppression including OCs, MDSCs, TAMs, Tregs, and pDCs, blockade of PD-1/PD-L1 pathway may confer an anti-MM effect by restoring the immune dysfunction. Early phase clinical trials in MM showed that blockade of PD-1/PD-L1 pathway alone does not achieve responses. Although combining PD-1 inhibitor with IMiDs (lenalidomide and pomalidomide) showed higher response rates in RRMM patients, clinical trials combining PD-1 inhibitors with IMiDs in MM are currently put on hold due to safety concerns.

On the other hand, anti-PD-L1 mAbs also show promising clinical benefit with acceptable safety profile in clinical trials of various solid tumors, leading to increasing interest in targeting PD-L1 in MM (174). Preclinical studies showed that treatment with anti-PD-L1 antibody induces no direct MM killing, but significantly restores the anti-MM activity of cytotoxic T cells or NK cells, suggesting that PD-L1 inhibitor might be a therapeutic partner with other anti-MM agents. Several combinations of molecules which either upregulate or downregulate expression of PD-L1 in combination with anti-MM agents are under evaluation (Table 1). Early phase clinical trials conducted with BCMA CAR-T therapy, HDAC6 inhibitors, and oncolytic reovirus in RRMM patients have shown preliminary promising results (175–177). Novel strategies targeting immune checkpoints and the OC-related pathway have also shown impressive results in preclinical studies. For example, the combination of RANKL and CTLA4 antibody enhances antitumor effect of lymphocytes (178). Blockade of RANKL pathway also augments the antitumor effect of PD1-PD-L1 blockade or dual PD1-PD-L1 and CTLA4 blockade in an animal model (179). Since RANKL inhibitor is now used in MM patients with bone disease, combinations with above agent represent potential novel therapeutic strategies. Finally, preclinical data combining CD38 with PD-1 and/or PD-L1 mAbs provides the rationale for clinical evaluation of these combinations. These various combination therapies may overcome primary and acquired resistance to anti-PD-1/PD-L1 therapies in MM.

An effective anti-MM immunotherapy not only relies on effective killing of MM cells themselves, but also on successfully restoring anticancer immune function. Immunotherapy targeting PD-1/PD-L1 pathway has revolutionized the treatment in several progressive solid tumors but is accompanied by immune-related adverse events in some patients. For anti-PD-1/PD-L1 immunotherapy to proceed in MM, it will be critical to investigate both the direct effects on tumor cells, as well as the impact on cellular- and cytokine-mediated immunosuppression in the MM microenvironment. Moreover, delineating molecular mechanisms regulating PD-L1 and PD-1 expression in the MM BM milieu will identify novel targets for potential therapeutic application.

## Author Contributions

Y-TT and S-FC review literatures and design and write this paper. Y-TT and KA critically review and edit the paper.

## Conflict of Interest Statement

KA serves on advisory boards Celgene, Millennium and Gilead Sciences and is a Scientific founder of OncoPep and C4 Therapeutics. P. Richardson is on advisory board of Celgene, Millennium and Johnson & Johnson. The remaining authors declare that the writing and research was conducted in the absence of any commercial or financial relationships that could be construed as a potential conflict of interest.
